# The Neurobiology of Behavior and Its Applicability for Animal Welfare: A Review

**DOI:** 10.3390/ani12070928

**Published:** 2022-04-04

**Authors:** Genaro A. Coria-Avila, James G. Pfaus, Agustín Orihuela, Adriana Domínguez-Oliva, Nancy José-Pérez, Laura Astrid Hernández, Daniel Mota-Rojas

**Affiliations:** 1 Instituto de Investigaciones Cerebrales, Universidad Veracruzana, Avenida Luis Castelazo S/N, Col. Industrial Ánimas, Xalapa 91190, Mexico; jim.pfaus@fhs.cuni.cz; 2 Department of Psychology and Life Sciences, Charles University, 182 00 Prague, Czech Republic; 3 Czech National Institute of Mental Health, 250 67 Klecany, Czech Republic; 4 Facultad de Ciencias Agropecuarias, Universidad Autónoma del Estado de Morelos, Cuernavaca 62209, Mexico; aorihuela@uaem.mx; 5 Neurophysiology, Behavior and Animal Welfare Assessment, DPAA, Universidad Autónoma Metropolitana (UAM), Unidad Xochimilco, Mexico City 04960, Mexico; mvz.freena@gmail.com (A.D.-O.); nancy.jopz@gmail.com (N.J.-P.); ashdez99.02@gmail.com (L.A.H.)

**Keywords:** well-being, neurobiology, learning, emotions, Panksepp, welfare

## Abstract

**Simple Summary:**

Animal welfare is the result of physical and psychological well-being and is expected to occur if animals are free: (1) from hunger, thirst and malnutrition, (2) from discomfort, (3) from pain, (4) to express normal behavior, and (5) from fear and distress. Nevertheless, well-being is not a constant state but rather the result of certain brain dynamics underlying innate motivated behaviors and learned responses. Thus, by understanding the foundations of the neurobiology of behavior we fathom how emotions and well-being occur in the brain. Herein, we discuss the potential applicability of this approach for animal welfare. First, we provide a general view of the basic responses coordinated by the central nervous system from the processing of internal and external stimuli. Then, we discuss how those stimuli mediate activity in seven neurobiological systems that evoke innate emotional and behavioral responses that directly influence well-being and biological fitness. Finally, we discuss the basic mechanisms of learning and how it affects motivated responses and welfare.

**Abstract:**

Understanding the foundations of the neurobiology of behavior and well-being can help us better achieve animal welfare. Behavior is the expression of several physiological, endocrine, motor and emotional responses that are coordinated by the central nervous system from the processing of internal and external stimuli. In mammals, seven basic emotional systems have been described that when activated by the right stimuli evoke positive or negative innate responses that evolved to facilitate biological fitness. This review describes the process of how those neurobiological systems can directly influence animal welfare. We also describe examples of the interaction between primary (innate) and secondary (learned) processes that influence behavior.

## 1. Introduction

Animal welfare is the result of physical and psychological well-being [1,2]. It is commonly inferred in each species from behavioral and physiological parameters that suggest normality under certain conditions [3,4,5]. For instance, the Farm Animal Welfare Council (FAWC) indicates with the Brambell report that for welfare to occur animals must be free: (1) from hunger, thirst, and malnutrition, (2) discomfort, (3) painful experiences, (4) to express their natural behavioral repertoire, and (5) from fearful and distressful events. Interestingly, from the neurobiological perspective well-being is not guaranteed by only providing continuous access to fresh water, food, shelter, comfortable resting areas, medicine, adequate environment, or the company of conspecifics. This is, in part, because well-being does not represent a steady state where animals can dwell throughout the day; rather it is a phase of motivated behaviors dependent of certain brain dynamics that function as emotional signals to indicate gains in biological fitness. Thus, by exploring the foundations of the neurobiology of behavior we approach our understanding of how well-being occurs in the brain and why animals express positive or negative emotions. With that in mind, we make clear that the reader of this manuscript will not find its applicability as a list of welfare activities to follow to the letter. Instead, we seek to generate awareness about how animal welfare implementation strategies may function (or not) as a result of modifications in the emotional brain systems. First, we provide the reader with a general view of the neurobiology of behavior. Then we introduce the levels of information processing and seven neurocircuits that evoke positive or negative emotions. We explore the influence of genetics and learning in the emotions evoked by those systems and the different phases of motivated behaviors. Finally, we discuss how the neurobiological approach may offer new opportunities of applicability and improvement of welfare programs.

To get started we must first recognize that all behaviors are expressions that result from the coordinated activity of the nervous system, which detects, processes, and responds to both internal and external stimuli, from the body or the environment, respectively. Detection of stimuli requires afferent pathways that act as entranceways to the central nervous system (CNS). Information in the CNS is processed according to its sensory nature and incentive value, both of which impact individuals depending on their age, sex, endogenous hormonal status and memories. Then, efferent pathways are activated, which may entail muscular, endocrine, emotional, cognitive and behavioral responses. We can generalize those mechanisms due to phylogenetic similarities among diverse species, where homologous brain structures, the same hormones and their receptors, and input/output pathways are responsible for similar behaviors that serve the same evolutionary goals (e.g., mating, survival). Thus, one can say that despite the diversity, animals share general mechanisms of afferent-processing-efferent information pathways in the nervous system that can be regarded as the foundations of the neurobiology of behavior. However, we must also recognize that evolutionary pressures may be different for each species, and in consequence, have resulted in specific adjustments and capacities that explain how some animals respond preferentially to certain stimuli more related to them, such as the scents or cues of potential sexual partners or predators.

**Figure 1:**
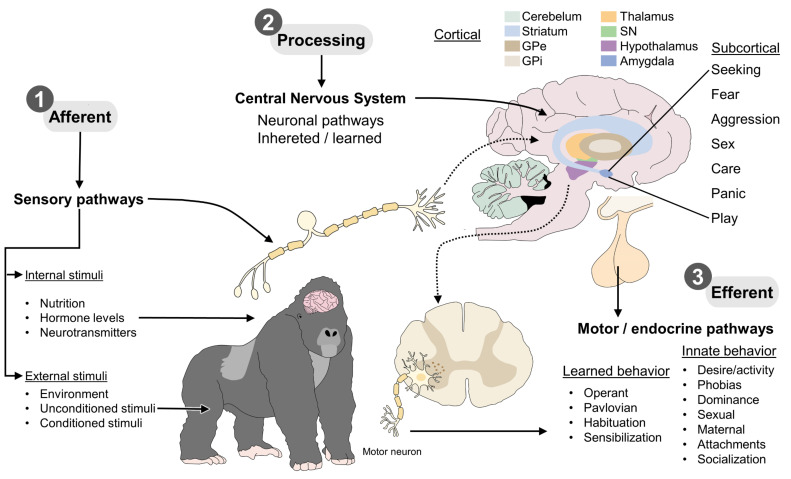
The big picture of how the neurobiology of behavior is relevant for animal welfare. All animals share general mechanisms of afference, processing and efference. The nervous system receives inputs from internal or external stimuli. Inputs are processed in various subcortical neurobiological systems to generate innate physiological and emotional responses that result in overt behavioral patterns. It can also be integrated into neurocircuits that employ learning to compare information stored in the form of memories to facilitate the expression of behavioral patterns. GPe: globus pallidus externus; GPi: globus pallidus internus; SN: sustantia nigra.

We hold the idea of former researchers that certain subcortical neurocircuits have been selected by evolution, and therefore, are inherited throughout generations supporting the display of specific emotional repertoires (that feel good or bad) to evoke behaviors that enhance biological fitness. However, we also recognize that not all behaviors are innate, but can occur as a consequence of secondary brain processes based on associative and non-associative learning. We provide examples of how by manipulating activities that influence innate or learned neurocircuits we can either generate or prevent emotional states and behaviors that directly impact animal welfare (Figure 1).

## 2. Levels of Information Processing

The neurobiological approach indicates that upon detection of the right stimulus certain subcortical neurocircuits will be activated and, in turn, will evoke emotions that make animals feel good or bad. This occurs via direct excitation, inhibition or disinhibition of other systems, influencing how an internal or external stimulus is perceived and processed. Neurocircuits that evoke emotions are phylogenetically stable and robust among mammals, which make emotional and cognitive differences among diverse species more of degree than of kind. This means that we can potentially use the neurobiological approach to understand how primary cerebral processes evoke similar emotions and behaviors in most animals. However, among species, there may be differences in secondary and tertiary brain processes which result in more complex responses that depend on associative learning and higher degrees of self-awareness, respectively. For example, the primary processes of sex (referred to as the *lust* system later on) evoke similar precopulatory (appetitive) and copulatory (consummatory) responses in all species with sexual reproduction. Accordingly, many different species may experience comparable degrees of “wanting” during the appetitive phase of an expected sexual encounter and comparable “liking” during the rewarding copulatory phase [6,7]. Although “wanting” may be expressed as pursuing or courtship behavior (as primary process), operant behaviors (as secondary process) or fantasies (as tertiary process), associated to the degree of learning, memory and consciousness, just as occurs in the expression of sexuality in humans [8]. Awareness of the levels of information processing is relevant to understand similarities among species and avoidance of anthropomorphic explanations.

The idea of the existence of primary brain processes as generators of basic emotions evolved in the early nineties thanks to the work of Jaak Panksepp (1943–2017), a worlds’ leading neuroscientist, who founded the concept of Affective Neuroscience and highly contributed to the study of human and mammal emotions [9,10,11]. Using electrical stimulation, pharmacotherapy and animal models of brain injury [12], he reported seven neurocircuits that function as basic neurobiological operating systems to generate basic emotions and innate behaviors. Panksepp referred to them as the systems of (1) SEEKING; (2) FEAR; (3) RAGE; (4) LUST; (5) CARE; (6) PANIC; and (7) PLAY [8]. To date, affective neuroscience is the basis for understanding the primary role of subcortical affective activation systems before cortical cognitive ones. Understanding the function of these systems is essential for cross-species and cross-cultural brain science [13].

Other neurobiological systems such as those that control hunger, thirst, and sleep are also relevant for welfare, although their participation is more related to basic homeostasis rather than emotional responses. Nevertheless, hunger and sleep can influence the response thresholds of other emotional systems. For instance, one study in humans [14] showed that hunger facilitates the expression of anger. Similarly, the lack of sleep or altered sleep patterns facilitates the expression of fear in animals [15].

**Figure 2:**
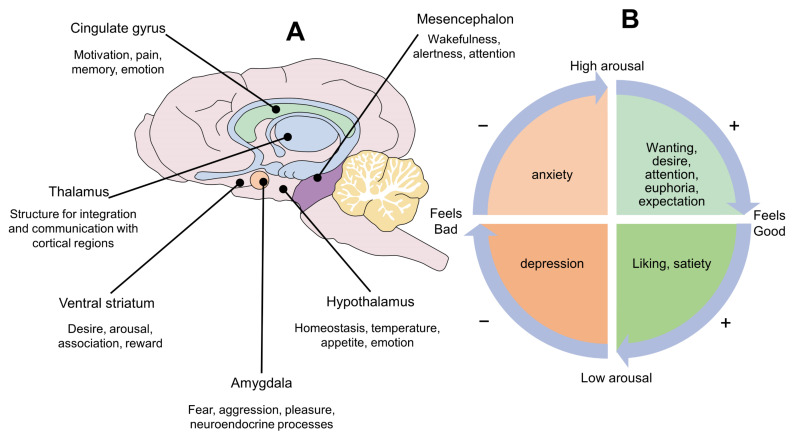
(**A**) Schematization and function of the limbic system in subcortical areas of the brain. Sagittal view of a dog’s brain (midline) indicating the main sub-cortical structures that mediate emotions. (**B**) Panksepp’s circumplex of emotions. The limbic system generates emotions that signal potential increases or decreases in biological fitness. Well-being is best achieved when animals experience emotions that move the vector towards the right quadrants. During the appetitive phase the vector moves to the right-upper quadrant (wanting), then during the consummatory phase the vector moves to the right-lower quadrant (liking). Emotions that move the vector away from the left side may be experienced as positive as well.

In general, the seven basic neurocircuits are concentrated in subcortical brain structures like the periaqueductal gray matter (PAG) of the midbrain, the medial thalamus, and hypothalamus. In turn, these structures have massive connections with higher cerebral regions known traditionally as the limbic system, including the amygdala, striatum, cingulate cortex, insula, hippocampus, and septum (Figure 2A). It is important to mention that, in this formulation, the neocortex has no main role in the basic neurobiological operating systems of behavior, but through a constant process of learning it can modulate their expression. Thus, as we will see later on, it is relevant to understand how animals learn and how we can use that knowledge to modulate neurocircuits that generate emotions.

## 3. Panksepp’s Circumplex of Emotions

Panksepp, Knutson and Burgdorf discussed the functional aspect of emotions as a part of a signaling pathway that potentially increases or decreases their biological fitness. As they state: “although animals are obviously not consciously computing their fitness, they are aware of their feelings at some level and respond accordingly, as evidenced by their behavior” [16]. They came up with the idea that all types of emotions (as behavioral drives) could be represented into a circle they called “the affective circumplex” (Figure 2B). The circle is divided into four quadrants. The two on the right symbolize positive emotions, whereas negative emotions are represented in the two left quadrants. Likewise, there are two upper quadrants that symbolize increased arousal, while a decreased effect is represented in the two lower quadrants. Panksepp and colleagues suggested a working model: (1) Exposure to stimuli that increase biological fitness evokes emotions that signal the potential benefit. Thus, the circumplex’s vector moves up and to the right. When that happens animals experience positive feelings and high arousal, which make them feel good and move more (e.g., euphoria, attention, high expectation). Similarly, emotions that depict a potential reduction in fitness produce a vector moving down and to the right. When that happens, animals experience positive feelings and low arousal, making them feel good and move less (e.g., relaxation, calmness, satiety). By contrast, emotions which signaling diminishes fitness produce a vector moving up and to the left. At this point, animals may experience negative feelings and high arousal, making them feel bad and moving more (e.g., anxiety, frustration), whereas emotions that signal reduction of potential increase fitness produce a vector moving down and to the left, which may evoke negative emotions and low arousal (e.g., depression).

All motivated behaviors have three sequential phases: (1) appetitive; (2) consummatory; and (3) post-consummatory. Those phases represent distinct but overlapping neurobiological states that, at a certain point, play antagonistic roles. For example, some brain structures are activated during the appetitive phase—generating desire and motivation—but are deactivated during the consummatory to post-consummatory phases, once satiety is reached [17]. According to the circumplex of emotions, well-being occurs every time an animal reaches the right quadrants, either by obtaining positive stimuli or avoiding negative ones. As explained above, the appetitive phase of motivated behaviors produce emotions that move the vector to the upper-right quadrant. This generates appetites, desire, attention and expectations, that energize the body. Animals move towards the source of interest and display other behaviors that serve to facilitate approach and consummation. Then, during the consummatory and post-consummatory phases the vector moves to the right-lower quadrant. Some authors have previously discussed the idea that well-being is more likely to occur if animals spend sufficient time in the right-upper quadrant before consummation [2]. In other words, animals should experience sufficient amount of “wanting” before they experience “liking”. Thus, animal welfare implementation strategies require identification of the animal’s current location within the four possible vectors and the choice of stimuli that may take it to the desired vector. Thus, awareness of the basic neurobiological systems of Panksepp may generate insights about animal welfare programs.

## 4. The Seven Basic Neurobiological Systems of Behavior

The seven neurobiological systems of behavior have the potential to produce positive or negative emotions by moving the circumplex’s vector upon modification of brain dynamics (Figure 2B). These systems have been explored using diverse techniques, such as behavioral analysis, administration of drugs [18], electrophysiology [19], and specific lesions in distinct regions (see [9] for further reading).

### 4.1. Neurobiological Systems That Evoke Positive Emotions

#### 4.1.1. Seeking

This neurobiological system is perhaps one of the most important for survival because it generates anticipation, expectation, desire, euphoria, search for resources and even causation [20]. When activated, the *seeking* system moves the circumplex’s vector towards the right-upper quadrant, producing arousal for all types of behaviors, accompanied by a certain level of pleasure. Not a pleasure like the one obtained after eating to satisfaction, but the feeling of anticipation of reward and arousal that animals may experience when anticipating reception of a desired outcome, such as food, a walk, socialization or a surprise. It also generates anticipation to sexual and other social encounters, which under certain circumstances may be more exciting than consummation of sex itself. The excitement for an imminent rewarding encounter or the joy felt when recognizing a partner or a playmate. In general, the *seeking* system generates the attention and motivation that are necessary in the appetitive phase of all motivated behaviors, which leads animals to search, approach, focus and obtain the object of their desire [19]. For example, when animals in captivity are offered food in circumstances that make immediate consumption difficult, the *seeking* system is activated which results in focusing on a task. If animals are focused and attentive to a reward it is less likely other systems will get activated. For example, one study showed that exposure to a homemade food dispenser hanging from the ceiling dramatically reduced fights of shelter dogs and increased play behavior during several days [21].

During the process of consummation animals trigger mechanisms of satiety. When that happens the *seeking* system reduces desire and arousal [2]. Accordingly, ad libitum access to the same food results in reduced interest and expectations. Animals with no expectations (i.e., for food) may look unmotivated and hypoactive, just like occurs quite often in captivity [22], indicating that the stimulus “food” is not longer sufficient to evoke activity in the *seeking* system. Under monotonous conditions other stimuli may become sufficiently powerful to activate either normal or abnormal activity of the *seeking* system, which in turn, may activate other emotional systems. Under abnormal high activity of this system animals express stereotypic movements, pacing, aggression, self-mutilation and compulsive behaviors, particularly in individuals with barren environmental conditions [2].

**Figure 3:**
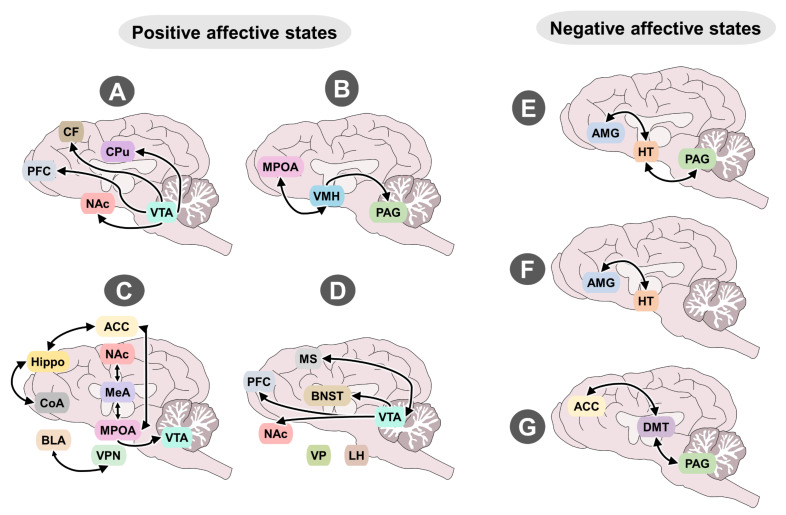
The seven basic behavioral neurocircuits according to Panksepp [9], schematized in a sagittal section of a dog’s brain. On the left side are the systems that evoke positive affective states: (**A**) seeking; (**B**) lust; (**C**) care and (**D**) play. On the right are the ones that evoke negative affective states: (**E**) rage; (**F**) fear; and (**G**) panic. Numerous brain regions are involved in these systems: amygdala (AMG), hypothalamus (HT), periaqueductal gray matter (PAG), anterior cingulate cortex (ACC), dorsomedial thalamus (DMT), prefrontal cortex (PFC), frontal cortex (CF), nucleus accumbens (NAc), ventral tegmental area (VTA), caudal putamen (CPU), medial preoptic area (MPOA), ventromedial hypothalamus (VMH), hippocampus (Hippo), cortical amygdala (CoA), basolateral (BLA), medial (MBA), and paraventricular nuclei (PVN), medial septum (MeA), bed nucleus of the stria terminalis (BNST), ventral pallidum (VP), and lateral hypothalamus (LH).

The *seeking* system consists mainly of dopaminergic neurons in the ventral tegmental area (VTA) of the midbrain [23] and its projections to the nucleus accumbens (NAc) in the ventral striatum and prefrontal (PFC) and frontal (FC) cortices (Figure 3A). Following exposure to relevant stimuli dopamine (DA) is released to the synaptic cleft where it binds DA receptors [24,25,26]. One study in primates showed that DA release occurred following unexpected rewards (food), but also following exposure to conditioned stimulus (a light) and anticipated reward (food). Interestingly, the activation of DA neurons diminishes when reward is not provided when expected [27,28]. Accordingly, the *seeking* system also generates positive emotions that signal concordance between expectations and rewards [29].

Neuroleptic drugs that block DA activity in the brain, such as acepromazine, haloperidol or azaperone, can modulate the activity of the *seeking* system. When that happens, animals behave less motivated and might even look like tamed. This is because low levels of DA activity reduce “wanting” and the capacity to focus on specific tasks, predict rewards or pay attention to cues that predict certain outcomes. By contrast, high levels of DA activity increase “wanting” and the capacity to associate stimuli and outcomes, which enhances expectation, attention, and appetitive behaviors.

In wildlife under human care, the appetitive phase evoked by activity of this neurocircuitry is used in operant conditioning programs, especially for a food reward. As mentioned by Grandin [30], encouraging the *seeking* system motivates learning when animals know they will receive a reward. This is frequently applied in sea lions during daily training sessions, in whom anticipatory behavior is considered an indicator of good welfare [31,32]. Another example is promoting a more complex and flexible environment for species such as Asian elephants (*Elephas maximus*). In them, the less predictability of food distribution encouraged foraging and an average walking of 15 km per day, a species-typical behavior that is beneficial to their physical and mental health by providing adequate space and resources [33].

This system also facilitates associations of stimuli, which is required for some types of learning. Consequently, animals can display motivation upon exposure to conditioned stimuli that predict a rewarding outcome [34]. For example, the use of clickers for training is a method of positive reinforcement where a sound of double-click, emitted from the device, is associated to a first-order conditioned stimulus. If this action is repeated enough, animals can be taught to show desired behaviors, serving as a rewarding stimulus itself [35].

#### 4.1.2. Lust

This neurobiological system allows animals to get excited, mate and reproduce. Its activation evokes emotions that move the circumplex’s vector towards the right-upper quadrant, producing high activation during courtship and pursuit of a partner. Then, after mating the vector moves to the right-lower quadrant, generating satiety to stop sexual behavior. This system also drives intrasex competition and some types of aggression in which individuals fight for access to potential mates [36]. This neural circuit is organized during the perinatal period but does not become fully activated until the onset of puberty, which is marked by increased levels of sex hormones [37]. It consists primarily of projections from the medial preoptic area (mPOA) to the ventromedial hypothalamus (VMH) and efferent projections from there to the VTA and PAG [38]. The appetitive phase of *lust* is produced when these structures, with their large amounts of receptors for steroid hormones like androgens and estrogens, are activated by oxytocin, melanocortins, DA, and noradrenaline. Then, during the consummatory phase those same areas are inactivated by the rewarding effect of serotonin, opioids, and cannabinoids [39]. The mPOA is considered a hub center of sexual motivation and performance. It can be activated by the medial amygdala through the accessory olfactory system, also known as the vomeronasal system, or by the piriform cortex through the main olfactory system. This means that natural scents from a sexual partner function as a powerful unconditioned stimuli to generate activity in the mPOA [40,41] but neutral odors that gained incentive value through paired associations with past reward can also activate the circuit. The function of this system depends on the levels of gonadal hormones and previous sexual experience. Therefore, veterinarians can control sexual desire and intrasex fights by preventing exposure to stimuli from receptive females, or by reducing gonadal hormones after gonadectomy. Without hormones the response threshold is increased, so most stimuli with sexual salience are no longer sufficient to evoke motivation. In the absence of sexual steroids mPOA neurons become incapable of releasing the neurotransmitter DA [42]. In cattle, when exposed to chronic stress due to nutritional, environmental, or health issues, the neuroendocrine stress response reduces the levels of luteinizing hormone, gonadotropin-releasing hormone, and estradiol impeding an adequate reproductive function [43]. This system is very robust but may not be active in undernourish individuals (Figure 3B).

#### 4.1.3. Care

This neurobiological system allows animals to maximize their biological fitness by providing care to the offspring. Its activation occurs following exposure to pups, which serve as stimuli to evoke positive emotions, moving the circumplex’s vector towards the right-upper quadrant, and producing high desire for the pups and experiencing reward when taking care of them [9]. The *care* system leads to the performance of specific behaviors in each species, including grooming, nest building, feeding, and defending the offspring from predators and danger.

In some cases, adults of one given species may express behaviors towards the young of other adults, and sometimes of other species. The neural circuit of care is shaped by the activity of three other subsystems. The first is responsible for identifying the newborn and involves the cortical amygdala, hippocampus, and anterior cingulate. The second is responsible for inhibiting fear evoked by infants and is formed by structures like the basolateral amygdala and paraventricular hypothalamus, which contain large amounts of oxytocin receptors. The third subsystem generates appetitive states and desire to care for a newborn. This includes the mPOA, NAc, and VTA [44], but some areas, such as the medial amygdala, seem to participate in all three functions [45]. Thus, cross fostering can successfully occur when the first subsystem is not highly selective to the own offspring. In females, the most effective stimuli for activating the *care* neurocircuit are vaginocervical stimulation caused by the birthing process, the reduction of progesterone, and the increase of estrogens. Other stimuli that can activate this neural circuit in males and females are copulation, moderate stress, and continuous cohabitation with offspring [46] (Figure 3C).

Maternal supervision has positive effects on the behavioral development of the offspring, reflected in greater social motivation and less fearful reactions. This is also associated with a lower probability of developing stereotypies during the socialization stage, a better adaptation to new environments, and, thus, a lower risk of injury due to fear-induced responses. In rodents and canines, maternal behavior is also essential for the behavioral development of offspring since puppies frequently groomed and cared for by their mothers have a greater capacity for learning and less fear of new stimuli [47]. Thus, allowing animals to express parental behavior during sufficient amount of time generates desire, keeps them attentive, and reduces anxiety. In addition, offers developmental benefits to the offspring.

#### 4.1.4. Play

This neurobiological system allows infant animals develop social and physical skills. Its activation evokes emotions that move the circumplex’s vector towards the right-upper quadrant, producing high activation and a certain level of pleasure. The neurobiological *play* system is activated in the absence of severe stress as spontaneous and rewarding behavior [48]. When active, it stimulates play in infants that can be characterized by five criteria: (1) the adaptive functions of play may not be evident at the moment it occurs; (2) play occurs because it is fun, a pleasant activity; (3) it resembles an exaggerated, incomplete form of certain adult activities; (4) it contains many repetitive activities performed with abundant variations, unlike stereotyped behaviors that are inflexible; and (5) play only occurs when animals are well-fed, comfortable, and healthy [49,50]. These conditions explain how the absence of play in infant animals indicates a severe alteration of their welfare.

In rodents, electrical activation of the *play* system evokes 50-kHz ultrasound vocalizations (USV) that can be associated with reward and euphoric states [49]. When infant rats are stimulated by tickling on the scruff of the neck and shoulders they also emit similar USV and play behavior [51], indicating that infants may experience tickling as a rewarding stimulus too. The *play* system requires integral thalamic pathways of the midline of the parafascicular and mediodorsal nuclei, which transmit sensory feedback to subcortical structures. Animals that have the entire neocortex removed are still capable of expressing normal social play, indicating that play (and the positive affect) is generated by subcortical structures. Cerebral electrical stimulation has evoked 50-kHz USV in other structures like the VTA, lateral hypothalamus, dorsal raphe, ventral pallidus, lateral POA, NAc, bed nucleus of the stria terminalis, medial septum, and prefrontal cortex [52] (Figure 3D). Vocalizations less than 50-kHz (22 kHz) are present during freezing behavior and occur as a response of the *fear* system in brain areas such as the amygdala or the PAG [53]. Therefore, high ultrasound vocalizations are postulated as a tool to evaluate the well-being of rodents and, possibly, other species [54].

The literature suggests that play is currently an indicator of the emotional state of animals. In domestic birds, the implementation of environmental enrichment increases the activity levels and reduces fear responses, improving the affective or emotional state of the animal [48]. However, physical enrichment is not always effective in all species. In calves, the addition of enrichment did not incite play; in their case, social coexistence (calves housed in pairs) provided a more pleasant living environment for the animals, so it is assumed that housing and social stimulation are two factors that lead to social play [55]. In domestic cats, social games (interaction with the owner or conspecifics) and the implementation of toys that promote activity and natural predatory behaviors keep them mentally stimulated. On the contrary, when felines lack this type of enrichment, their behavioral repertoire is limited, and they are predisposed to present behavioral pathologies that affect their health and coexistence with their owners [56].

### 4.2. Neurobiological Systems That Evoke Negative Emotions

#### 4.2.1. Rage

This neurobiological system allows animals to defend their resources and offspring. Its activation evokes emotions that move the circumplex’s vector towards the left-upper quadrant, producing high activation and a certain level of displeasure. Activation of the *rage* system evokes emotions and behaviors that force others to do something (or cease doing something) that compromises resources or the welfare of the offspring [57]. In other words, the aggression system promotes several behaviors that allow securing resources, enhance success of mating or access to a territory, an essential trait for protection and survival [58].

Animals can display rage in different forms, which is normal in certain contexts, such as competitions, or when defending resources, and because of frustration or fear, but also as part of the repertoire of predators. The expression may depend on the intensity of activation of the system, going from mere warning displays (i.e., vocalizations) up to explicit aggressive behavior. In general, aggression can be classified in two types: defensive and predatory. The first is said to be emotional, difficult to control voluntarily, and marked by multiple signs of autonomic activity, whereas predatory aggression is more controlled, has no signs of emotion, and has little autonomic activity [59]. The neural circuit of *rage* is formed by connections of the dorsal and medial PAG, medial and lateral hypothalamus, and amygdala. During episodes of defensive rage, the dorsal PAG receives stimulation relayed from the amygdala via the medial hypothalamus. In predatory aggression, the ventral PAG receives stimulation from the lateral hypothalamus [57]. Certain chemicals facilitate the expression of aggression in the presence of triggering stimuli. For example, high levels of testosterone, substance P, norepinephrine, glutamate, acetylcholine, and nitric oxide synthase [60]. However, the influence of chemical mediators in the initiation of this system depends on the species, environment, and seasonality, as seen in caged-housed common pheasants (*Phasianus colchicus*), where total plasma testosterone levels were influenced by the breeding season but did not differ between aggressive and non-aggressive males [61]. In some cases, the antagonists of these substances inhibit aggression or reduce affective appetitive states, as occurs with propranolol (beta blocker of noradrenergic receptors). In males, gonadectomy reduces testosterone levels, thus raising the response threshold of defensive aggression (e.g., territorial, intersex, dominance). In addition, increases in cerebral serotonin are also associated with a raising of the detonator threshold of aggression, which explains how some antidepressants that increase serotonine availability may function to decrease certain types of emotional agression (Figure 3E). For example, in dogs, although some studies have shown no relation between serum serotonin and aggressive, fearful or impulsive canines [62], serotonin treatments have been suggested as a potential tool to monitor behavioral disorders [63]. Odore et al. [64] reported this in a case study of eight dogs diagnosed with dominance-related aggression by a behaviorist expert, receiving a serotonin reuptake inhibitor for six months. The subjects showed a significant decrease in aggression and circulating serotonin levels, associating these with a clinical improvement and an alternative to aid in this public health issue. In other species, like the zebrafish, pharmacotherapy using serotonin antagonists abolished aggression [65].

This rage or aggression response to events that compromise animal welfare is frequently observed in production units where good husbandry conditions are absent. In the case of pigs, aggression towards conspecifics (tail biting) is common. This is attributed to an improper diet, overcrowding, and lack of substrates or options that allow the animal to maintain a positive mental state [66]. On these farms, studies like the one by Ko et al. [67] have shown that the addition of hemp ropes or rubber chew toys reduces piglets’ post-weaning aggression and the presence of skin lesions and ear-biting up to 3.3 times. Additionally, salivary parameters (cortisol, chromogranin A and α-amylase) diminished on piglets. This suggests that the *rage* system decreased its activity when the *seeking* and *play* systems were active. This does not only apply to mammals. The reduction of territorial aggression has also been reported in captive fish such as black rockfish (*Sebastes schlegelii*) and fat greenling (*Hexagrammos otakii*) when their environmental conditions are improving, resulting in good welfare in aquaculture [68]. Interestingly, some studies such as Woodward et al. [69] have found that housing enrichment increases aggression in the zebrafish (*Danio rerio*) and affects their growth due to its natural territorial behavior to defend food resources in potential spawning sites. Therefore, to understand this system is necessary to consider the species, the stage of life (i.e., juvenile or adult), and the environment of animals.

#### 4.2.2. Fear

This neurobiological system allows animals to get away from dangerous situations. Its activation evokes emotions that move the circumplex’s vector towards the left-upper quadrant, producing high activation and a high level of disconfort. The expression of fear is an adaptive response and therefore facilitates biological fitness of the species. Upon exposure to an intense dangerous stimulus, the *fear* system prompts the animal to flee or freeze, in order to avoid serious injury or death [70]. Jaak Panksepp explained how laboratory rats will freeze upon exposure to cat’s fur, despite the fact they have been bred and maintained isolated from their natural environment and free of predators during many generations. In this case the scent of a predator (cat) functions as an unconditioned stimulus capable of activating the circuit of fear without the need of previous learning [9,71,72]. In newborns, this system is ready to respond with emotional states that generate immobility, fright and, in some precocial species, flight. In addition, explorations of the *fear* neurocircuit have used electrical stimulation of the brain to evoke fear responses even though no threatening stimulus is present. When the applied stimulation is low, the responses tend to be of the immobility (freezing) type, but when electrical stimulation is more intense it can generate “fight-or-flight” responses [73]. For this reason, it is widely believed that the varied expressions of anxiety indicate a slight state of activity of the *fear* system, while terror is a consequence of greater activity. This neurocircuit includes central and basolateral zones of the amygdala near the anterior and medial hypothalamus, and from there towards the dorsal region of the PAG [73,74]. Since the amygdala expresses large amounts of GABAergic receptors, benzodiazepine agonists can reduce the affective states and the behavioral responses associated with fear (Figure 3F).

The absence of fear is one of the five animal freedoms to assure animal welfare [75]. In farm animals, fear caused by aversive human-animal interaction has an accumulative effect that, in the long-term, affects their handling, health, welfare, and productivity in terms of milk and meat byproducts [76], where animals, such as cattle, can be exposed to this system during slaughter, representing a severe welfare hazard [77]. Recent studies with horses have shown that the reduction of the fear response can be learned through a social transmission effect, where young horses exposed to habituated adult conspecifics reduced fear-related behaviors and heart rate values [78]. In sheep, novel measures such as infrared thermography have been implemented to assess the fear of humans through voluntary approach tests. Thus, this technique could be useful to determine negative states in production animals [79].

On the other hand, although fear is frequently connoted to a negative state [80], in wildlife species, particularly those that are part of reintroduction programs, fear of humans and absence of habituation to humans is a key element and a natural behavior required to increase the success of reintroduction protocols [81]. In the case of the Javan slow loris, a species that is critically endangered due to illegal trade, Campera et al. [82] have shown that habituation to humans increased the possibility of death and being re-captured by traders, reducing the level of alertness of the animals, a trait that is necessary to avoid predation. This effect does not only cause problems with the animals but also arises human-wildlife conflicts that cause consequences for the animal. An example is ungulates, in whom their habituation to human presence induces excess herbivory and damage to the habitat, ecosystem, or threatening human health [83]. One way to reverse this effect and the neurobiology of the *fear* system is through aversive conditioning to maintain the value of the ecology of fear in wildlife. However, intentionally instilling stress and fear in animals drives ethical issues in the management of wild animal populations [84].

#### 4.2.3. Panic

This neurobiological system allows animals to maintain family bonds. Its activation evokes negative emotions that move the circumplex’s vector towards the left-upper quadrant, producing high activation and social pain. The *panic* system does not produce emotional states of fear, but rather, negative states associated with the loss of a social bond [85]. When social bonds are affected by death or separation, the *panic* system generates negative states marked by distress and expressed mainly through vocalizations [49]. The initial affective bond in all mammals is the mother-infant relation that begins at birth [86]. A warm, secure maternal relation generates secure, emotionally stable young. This system is especially valuable in altricial animals where the survival and welfare of their vulnerable newborns depend on their capacity to emit vocalizations of distress that attract the attention of the mother, who represents the infant’s source of food, thermoregulation, care, and protection [87]. This affective bond is strengthened through lactation and cohabitation, but when the *panic* system is hyperactive, it generates separation anxiety at the slightest sign of the absence of the attachment figure. By contrast, when the system is hypoactive, it prevents the formation of affective bonds. Experimental studies show that distress vocalizations can be generated by electrical stimulation of the anterior cingulate, dorsomedial thalamus, and PAG [88,89]. The *panic* system reduces its activity following administration of opioids, oxytocin, or prolactin, as they reduce distress vocalizations in animals [85]. This is supported by the fact that social contact produces reward by releasing endogenous opioids, oxytocin, and prolactin (Figure 3G). Young animals that experience panic may find consolation in social contact and petting.

The activation of the *panic* system is an essential element for the welfare of wildlife, production, and company animals. As Mellor [90] mentions, encouraging the positive side of this system promotes bonding with conspecifics or social groups to provide companionship and protection. Likewise, bonding implicates the communication between animals and humans and the improvements that can derive from it. For example, domestic dogs are a social species by nature. When they develop an attachment to their owner, distress behaviors are observed and represent a serious impact on the welfare of the animal but also the human-animal interaction [91]. Separation distress in animals can be considered a disorder equivalent to phobias and panic attacks in people, with parallel consequences when an individual cannot cope with its environment (e.g., self-mutilation, aggression, physiological alterations, elimination abnormalities, vocalization, destruction, and even depression) [92].

## 5. Genetic Bases of Behavior and Its Relation to Welfare

**Figure 4:**
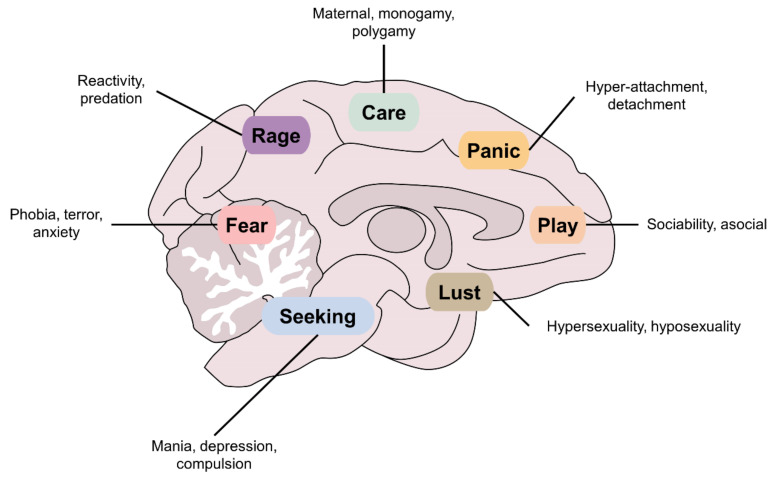
Examples of genetic influence on animal behavior. The basic neurobiological systems represent the phylogenetically robust neural substrate for the expression of behaviors. Selective mating can generate individuals with enhanced sensitivity to a certain kind of stimulation, leading to overexpression of the behavior involved. Likewise, selection can decrease sensitivity to stimuli, thus reducing the probability of a certain behavior being expressed. Those manipulations can be positive or negative to animal welfare.

We must consider the basic neurobiological systems of emotions as robust and phylogenetically stable, but at the same time susceptible to heritability. Thus, within a given population some individuals may express emotions with high intensity, whereas others may barely respond to the right stimulus, which means not all individuals will experience positive or negative emotions with the same intensity. For example, the *care* system evokes maternal behavior and positive affect following exposure to pups, but not all dams respond with the same intensity. Experiments in rats have shown that high responder mothers (i.e., high pup licking) give birth to high responder daughters (when adults). Inheritance of a neurocircuit corresponds to the expression of certain genes in specific brain areas that may facilitate neurotransmission within that particular area (Figure 4). For instance, monogamous females show better maternal behavior and more oxytocin receptors in the NAc and prefrontal cortex compared to promiscuous species; while the monogamous males also express better paternal behavior and more vasopressin receptors in the septum and ventral pallidum [93,94]. Lim et al. [95] showed that promiscuous rodents increased their paternal behavior after treatment with viral vectors that replicated the receptor coding gene for vasopressin in the septum and ventral pallidum to an exaggerated degree. This indicates that mutation of one single gene affects the expression of the behaviors generated by the *care* system and that some individuals may be more susceptible to experience well-being if allowed being parental. The environment also plays a fundamental role in activating or silencing genes through epigenetic mechanisms. For example, daughters of low responder dams can become high responders and even modify the expression of the related gene (Grm1, metabotropic glutamate receptor) if they are raised by foster high responder mothers [96,97]. This indicates that pups also learn parental behavior from their parents.

Inheritance also affects the sensitivity of the *seeking* system. At normal levels this system evokes attention, expectation and desire to explore, but some animals may be prone to display abnormal behaviors such as compulsivity. Dodman and colleagues [98], used genome-wide association analyses to compare genes of Doberman pincher dogs affected by canine compulsive disorder (CCD) vs. unaffected, and reported differences in a locus of chromosome 7, where the most significant single-nucleotide polymorphism was located within the neural cadherin-2 (CDH2) gene. CDH2 mediates synapsis function in dopaminergic areas such as the ventral midbrain and prefrontal cortex [99]. Interestingly, it has been reported that some Belgian malinois dogs typically show a circling behavior (one kind of obsessive-compulsive behavior) when placed in a confined space. Those compulsive dogs tend to show better work performance than dogs without the circling behavior and also express differences in CDH2 [100]. This evidence clearly indicates the heritability of the *seeking* system, so artificial selection of animals with high levels of attention and motivation also selects neurobiological features associated with compulsion.

Something similar occurs with the *rage* system. Dmitri Belyaev and colleagues [101] studied the heritability of aggressiveness over 35 generations of domesticated foxes (a total of 45,000), over 40 years. First, the foxes were aggressive towards humans. However, less aggressive ones were selected to be bred with other non-aggressive foxes. The resulting pups were also selected for extended breeding based on their docility. New generations of foxes began to show signs of domestication similar to dogs, such as screeching for attention, hand licking, tail wagging, and barking. They also began to express floppy ears, shorter legs, shorter and curvier tails, and colorful fur. Their skulls were narrower and their snouts shorter than those of non-domesticated foxes. The females began to go into heat twice a year, instead of once like undomesticated foxes. Domesticated pups opened their eyes sooner and showed fear responses much later than undomesticated pups. In domesticated foxes, it was found a decrease in the production of adrenal hormones and more serotonin. Finally, more than 40 gene differences were found between domesticated and aggressive farm foxes and more than 2700 gene differences between farm and wild foxes. Accordingly, that study showed that domestication of foxes resulted in less responsive *rage* and *fear* systems, which correlates with certain foxes’ phenotypes, like coat color (Figure 4). Similarly, Ortiz-Leal et al. [102] suggest that these phenotypic changes that resulted in domestication go beyond external features, changing neuroanatomical aspects such as the structure and functionality of the accessory olfactory system (AOS). In foxes, the AOS has a higher degree of development when compared to the domesticated dog.

Coat color in cats also predicts aggressive behavior. It has been reported that orange, gray-and-white, and black-and-white coats in cats do offer some evidence that supports their greater aggression towards humans [103]. The coat color, docility, expressions of play, modified adrenal function, and reduced cranial-facial dimensions are some of the many characteristics that accompany domestication. It has been discussed that part of the origin of these differences reflects alterations of cells in the neural crest, where melanocytes, adrenal glands, teeth, and facial bones all emerge. For instance, Singh et al. [104] showed that color and cranial-facial dimensions were modified after 64 generations of rats selected for docility or aggressiveness.

The importance of genetic mechanisms in the study of animal behavior lies in their potential to improve our understanding of how activation of the brain mediates animal welfare, and brain activation depends on many biological, molecular, neural and genetic mechanisms that result in phenotype and behavior. Accordingly, we must understand that primary-process of behaviors require an innate factor, a genetic influence, and the environmental context. However, we must keep in mind that the resulting emotions and behaviors can also be affected by learning, experience, and other elements that alter the development of innate response [11].

## 6. Innate Responses That Impact Welfare

**Figure 5:**
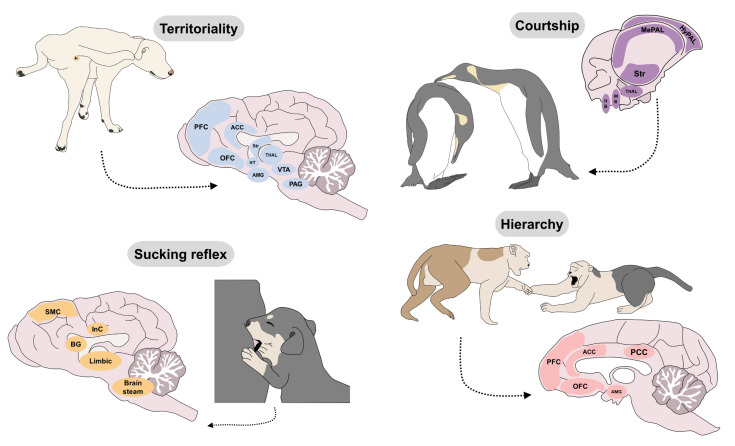
The basic neurobiological systems of behavior evoke developmentally-dependent innate responses. For example, delimiting a territory by marking it with urine reflects the systematic activity of the rage/seeking/lust systems and begins at puberty. Courtship behavior, as in the case of the male penguin that sticks out its chest, raises its beak, and tilts its head back (lust/care systems). The suckling reflex in puppies (seeking, panic) and the establishment of dominance hierarchies in wolf packs (rage/seeking) are other examples. In every innate response, the main cerebral structures involved in the processing of its respective integration is schematized in the brain. ACC: anterior cingulate cortex; AMG: amygdala; BG: basal ganglia; HB: hindbrain; HT: hypothalamus; HyPAL: hyperpallium; InC: insular cortex; MB: midbrain; MePAL: mesopallium; OFC: orbitofrontal cortex; PAG: periaqueductal gray; PCC: posterior cingulate cortex; PFC: prefrontal cortex; SMC: sensorimotor cortex; Str: striatum; THAL: thalamus; VTA: ventral teg-mental area.

The existence of genetically determined neurocircuits of behavior supports the idea that all mammals, and perhaps other species as well, will respond with emotions and specific patterns of behavior upon encounters with natural stimuli that activate those circuits. As explained above, such emotions function as biological signals of biological fitness. Thus, when an animal is curious, playful, excited and affectionate is expressing innate emotions that feel good because the resulting behaviors represent an advantage in their survival, reproduction and transfer of their genes (Figure 5). For example, since piglets are known as very curious species, enrichment programs are easy to implement in these animals. As de Souza et al. [105] have stated, environmental enrichment using flavored sisal ropes (natural, vanilla, and coffee flavors) in 66 piglets with 35 days of age elicited positive reactions, curiosity from the animals (21.4%), and preference values towards the vanilla flavor (63.2%) [105]. Likewise, in the same species, in 297 fattening piglets, the less fearful reaction, quicker approach latency, and longer duration of contact with different environments, including human contact and novel object exposure, is an element usually associated with good welfare. However, the relation of high/low levels of motivation to explore and its positive or negative valence into the affective state has not yet been established and needs further study in all species [106]. This trait could be helpful not only in production units but also in a laboratory setting. Piglets can be trained using their investigative nature to reduce stress, aversive behavioral reactions and avoid bias studies [107]. On the contrary, when an animal is fearful, aggressive and depressed is expressing innate emotions that feel bad to evoke behaviors that prevents them from dying, getting hurt or being left alone/unprotected.

The term “innate” refers to “patterns of movements that do not require previous experience for their proper execution” [108] and indicates that individuals are born with the capacity to express those emotions and behaviors upon exposure to the specific triggering stimulus. However, some responses referred to as innate may not be expressed immediately at birth, but later, when the individual reaches infancy (*panic*, *aggression*, *play*), puberty (*lust*), or adulthood (*care*). This means that the innate behaviors that emerge from the basic operating systems depend on ontogenic processes.

It is important to clarify that, innate behaviors are not exactly the same among individuals of a given species [109]. There is evidence indicating that even simple innate behaviors show intra-individual and inter-individual variations [110]. For example, primates show high levels of intra-individual behavioral flexibility, which is a component of adaptability to the environmental and genetic mechanisms [111]. Likewise, some behaviors (e.g., antipredatory ones) can differ in the same individual exposed to the same stimulus at different times. Hence, some authors suggest that the study of inter and intra-individual differences associated with innate behavior must consider the presence of a group that may result in an innate response influenced by mimicking or social learning [112]. In rodents and birds, vocalizations are considered an innate behavior that develops in the absence of auditory experience and consists of diverse neural circuits. Contrary to birds, in mice, its generation is independent of the motor cortex, where decorticated males can still generate ultrasound vocalizations [113]. In the case of birds, vocalizations are considered innate elements; however, they are the result of a socially learned communication that differs between species, depending on the environment, and the intention (e.g., courtship, antipredatory) and the result of cumulative cultural evolution [114]. This could be the reflection of a favorable environment for the animals or one that limits their behavioral repertoire (Figure 5).

The expression of an innate behavior requires exposure to unconditioned stimuli that activate afferent sensory pathways with no need of prior learning. For example, in many animal species, the sense of smell responds unconditionally to olfactory cues generated by pheromones [115]. Wyatt [116] defines pheromones as chemical substances in individual compounds or combinations of molecules that function as communication signals among members of the same species. While they may participate in all motivated behaviors, they are known to function mainly in reproductive and fear behaviors. They are released into the external environment. Then, other individuals can detect them through the vomeronasal organ (VNO) and the olfactory epithelium (MOE), which consecutively prompt affective responses that can trigger motivated behaviors [115,117]. In other words, pheromones are a type of unconditioned stimuli that can specifically activate neurocircuits to generate innate responses or patterns of behavior. In dogs, their ability to detect a predator’s location through chemical cues in feces, urine, or anal gland secretions is considered an innate and evolutionary response to avoid danger (Figure 5). One study explored the responses of 82 dogs exposed to water, brown bear (*Ursus arctos*) and Eurasian lynx (*Lynx lynx*) scents. Dogs exposed to the smell of bears spent less time within the scent and their heart rate increased about 30% above baseline. Therefore, this reaction to odors may have an influence in the degree of activation of emergence systems (such as the autonomic nervous system) [118], and recent therapeutic options use pheromones to reduce stress-related behaviors in companion or shelter dogs [119].

In general, innate responses (*seeking*, *play*, *fear*, *panic*, etc.) occur naturally because they are useful to animals in their natural environment, regardless of whether they feel good or bad. However, all innate responses can be enhanced or decreased by means of environmental enrichment and learning mechanisms. Understanding this may be useful to achieve animal welfare.

## 7. How Learning Influences Animal Welfare

At birth, most mammal neonates express distress vocalizations (*panic* system) under various conditions, including hunger, cold, or a lack of security. Females are especially sensitive to these vocalizations in the postpartum period and respond with behaviors indicative of maternal care. The mother-infant interaction is essentially identical in terms of its behavioral repertoire in all couples of the same species. It obeys to the activity of the basic neurobiological systems of panic for the offspring, and care for the mother. However, as time passes infants learn through cognitive mechanisms that their mother will come when she hears their vocalizations. In consequence, infants modulate the frequency, latency, and intensity of their vocalizations. Similarly, with time, or after several maternal experiences, females learn to respond to, and care for, their infants more efficiently. In this framework, learning can represent a behavioral modification posterior to an experience. If that behavioral change endures over time, it is assumed that storage has occurred in the form of memory. What animals learn can have a direct impact in their well-being. Sometimes, learning may either reduce or enhance the effects of emotions and behaviors.

Learning is understood as the principal means of acquiring information, while memory is defined as the brain’s ability to decode, accumulate, order, and recall information generated by the positive and negative experiences that an animal perceives throughout its lifetime [120]. Physiologically speaking, memory stores experiences as traces that can be recapitulated thanks to alterations in the perceptibility of the synaptic transmission from one neuron to another. The variations produced lead to the formation of new pathways known as memory traces. Once established, traces can be stimulated to reproduce the memory [121]. This means that, memory traces can influence the basic neurobiological systems of behavior, facilitating or inhibiting its activation.

### 7.1. Making Memories

We must note, however, that not all experiences are stored in the form of memories. Shifting the short-term memory to long-term relies on repetitions or intensity of the experience and it requires four phases that occur in the synapses: (1) The synaptic trace must be generated; (2) stabilized; (3) consolidated; and (4) maintained [122]. Thus, we can say that only those memory traces that are metabolically maintained will persist as long-term memories, whereas those traces that fail to be consolidated and maintained will fade away after a short period of time. For example, lets imagine the infant that upon separation of the mother responds with distress vocalizations. During the first episodes, the intensity of panic and the frequency of vocalizations are set by default, depending on innate mechanisms. Emission of vocalizations associated with mother’s attention will generate a trace in neurons that form part of the *panic* and *seeking* systems, activated when an animal lives the experience. The cellular correlates of memory formation have been explained as follows [123]: Within the first minute post-experience, activity of the glutamatergic receptors N-methyl-D-aspartate (NMDA) triggers the entrance of calcium into the dendritic spines of the postsynaptic neurons. There, scaffolding proteins like actin are disassembled to facilitate the mobilization of GluA1 AMPA type glutamatergic receptors from the cytoplasm to the membrane of dendritic spines. The presence of more GluA1 AMPA receptors, in turn, fosters the temporal excitability of the synapse. Stabilization of the trace occurs approximately 20 min after the experience as calcium levels in the dendritic spines continue to rise. During stabilization, actin is polymerized, permitting the capture of the receptors that have been mobilized in the first minutes. Then, the trace can be consolidated 2-4 h after the learning experience through the participation of metabotropic glutamate receptors (mGluR) as calcium continues to enter increasing intracellular secretion of the brain-derived neurotrophic factor (BDNF) with the onset of local synthesis of proteins and their transcription. In addition, ubiquitin-proteasome (UPS) enzymes complex degrade those transcription and translation repressing proteins. Then, if the process is repeated many times or if it is emotionally intense, maintenance of the trace occurs to perpetuate the intracellular mechanisms that occurred in the dendritic spines. During maintenance there is production of the MZ protein kinase, and the GluA2 AMPA type glutamatergic receptors are released to replace GluA1. Although these mechanisms have been mainly explored in laboratory conditions, they are likely to occur as such in living animals. Understanding the processes of learning and memory is relevant because long-term memories (good or bad) can last an animal’s lifetime.

In some species, memory for certain events dictates how animals react to their environment in the future. In the case of horses, they respond differently according to the valence of past interaction. When exposed to conditions associated with past negative experiences, animals show ears held backward and right hemisphere activation, signs of a negative mental state [124]. Likewise, they can remember the facial expressions of humans and can adjust their behavior accordingly [125]. These examples show that the consequences of a negative experience are not limited to the immediate period. It can influence the behavioral response and could affect their welfare or inter- and intra-species social interactions.

### 7.2. Types of Memory

After a given experience memory traces can be active during a fraction of a second (sensory), during few minutes (short-term) or for a lifetime (long-term). This means that depending on how intense or how many times occurred an association, stimuli with a learned outcome can generate emotional responses relevant to animal welfare. As it could happen in dogs exposed to familiar olfactory signals that elicit memories in the presence of those scents [126]. Short-term memory allows the animal to do follow-up on the relevant information, update it, and use it to modulate behavior at the same instant. It gives logical continuity to social interactions in the present, allowing individuals to know what just occurred in the environment and what strategies they need to adopt. Thus, contingency and contiguity between a stimulus and its outcome can be associated ipso facto to either strengthen or weaken future learned responses via Classical or Operant conditioning. Many phobias, aberrant behaviors or potential behavioral treatments depend on these mechanisms.

### 7.3. Classical Conditioning

Classical (Pavlovian) conditioning is the simplest kind of associative learning. It is important because by associating stimuli with concurrent physiological responses, an animal can develop the capacity to anticipate events and generate adaptive responses. The key aspect of this for animal welfare may be the emotional and behavioral response associated with fear or euphoria. The basis of classic conditioning is the existence of unconditioned stimuli (US) that generate unconditioned responses (UR); that is, natural stimuli that produce natural physiological responses in the fear (or other) system with no need for prior learning. However, neutral stimuli (NS) that do not normally produce a natural response may begin to do so if they come to be associated with a US through contingency and contiguity. Hence, after various associative repetitions, an NS is transformed into a conditioned stimulus (CS) capable of generating a conditioned response (CR) that are physiological and affective in nature [123]. In this scenario, the *fear* system can be activated by stimuli that did not have the capacity to activate it before conditioning. The natural or artificial environments of animals contain diverse sounds, lights, odors, and manipulations that can function as CS and generate CR of fear, aggression, joy or sexual desire. Classical conditioning can last a lifetime, is involuntary, and occurs unconsciously.

### 7.4. Operant Conditioning

This type of learning is generated by the capacity to associate one’s own behavior with a possible result. When an animal randomly “does something” and immediately afterwards “something happens” an association occurs between the behavioral response and the result. For example, if a certain behavior leads to a reward, it is likely to be repeated; by contrast, behaviors that do not produce a reward will decrease in frequency [123]. Likewise, if a given behavior results in some kind of punishment, it is likely to decrease in frequency and probability. In this type of conditioning, behavior is associated with the presence or absence of reinforcers and punishments. For example, if a newborn vocalizes and the mother responds immediately with her presence, the young one learns the consequences of its behavior and will try to vocalize more often to draw its mother’s attention under various circumstances. But if the mother does not come, it is highly probable that the newborn will gradually reduce the frequency of vocalizations. It is important to note that contingency and contiguity are two key factors involved in behavior and its consequences, and that they are necessary for conditioning to occur.

### 7.5. Habituation

Habituation is considered the simplest form of non-associative learning and it is useful to reduce undesired behaviors (whether normal or not). It is defined as a reduction in the arousal or response to a stimulus due to gradual, repeated exposure to it that is not attributable to sensory or motor fatigue, such that tolerance for the disturbance increases [123]. This is a long-term, stimulus-specific process that does not involve reinforcement, but depends on three features: the nature and frequency of the stimulus, and the regularity of exposure. Habituation differs from the extinction of a behavior, in that the aim in the former is to eliminate or decrease a response that is part of an innate behavior of the species, not to eliminate a previously conditioned response, as occurs in extinction [127]. This means that animals can generate habituation towards natural stimuli that activate the basic neurobiological systems, but they cannot undergo extinction. For example, animals can be habituated to reduce their response upon exposure to stimuli that trigger activity of the *fear* system, like noisy sounds, odors from other animals, or humans. They cannot extinguish their capacity to feel fear. Nevertheless, there are three important phenomena that must be considered when animals are habituated: (i) stimulus generalization, which means a possible rapid habituation to a new stimulus that is similar to a previous one; (ii) spontaneous recovery, or a rebound under prolonged periods between re-exposures; and (iii) dishabituation, or the reestablishment of habituation [128,129].

All animals, especially pets, become habituated to the presence of humans and associated visual and auditory changes in their surroundings [130]. This occurs, as well, in ruminants in production units and in horses and other riding animals that become habituated to the act of being mounted [131]. Mills et al. [127] mention that habituation to the presence of humans and other potentially aversive stimuli during ontogeny reduces the probability of animals presenting alterations in adult life. For example, thunder-phobic dogs that are exposed gradually to recordings of the sounds of storms may become habituated to that noise, though one must keep in mind that an animal may fear not only the sound but some related factor that triggers their reaction [132]. In general, habituating animals entails benefits for their welfare. For example, habituating donkey foals to, and training them for activities like loading and transport reduced the stress they exhibited [133]. In addition, adaptation to the presence of, and handling by people has been shown to have favorable results in production units [80]. A study that compared female Martina Franca dairy donkeys habituated or not habituated to the milking parlor demonstrated that 40 unaccustomed animals had more stress-related behaviors and 25-70% lower milk production. Results were attributed to reduced oxytocin concentrations due to increased heart rates and vasoconstriction [134]. Habituation prior to milking has also been shown to reduce stress and increase yields in species like water buffalo [135]. In the case of zoo animals, habituation and other methods of learning are widely utilized to foster human-animal relations and interaction with the environment. For example, habituating ring-tailed lemurs to their caregivers, to changes of stations inside their enclosures, and to the presence of visitors, impedes them from presenting certain stereotype behaviors [136]. However, it is important to consider that habituation does not always produce positive features in animal, as mentioned in the *fear* system section. Work on ecotourism programs where anthropogenic activity can produce habituation has been shown to reduce vigilance levels in Nubian ibexes (*Capra nubiana*) that can impact their survival and biodiversity [137]. Likewise, reintroduced rhinoceroses that are habituated to human care generated reductions as high as 0.19% in their alert reactions to the presence of human with fewer affiliative behaviors, abnormal feces, and less behavior related to positive emotions post-reintroduction [138]. These examples suggest that habituation reduces the reactivity of the *fear* system. This may bring advantages or disadvantages to animal welfare, depending on the context.

### 7.6. Sensitization

This is the opposite of habituation; that is, when a stimulus evokes a more intense response than before. For example, certain animals react in an exaggerated manner to the presence of other animals. Phobias, in a certain sense, can result from sensitization to a stimulus. All animals can be habituated or sensitized depending on the intensity of a stimulus. Intense stimuli tend to cause sensitization; that is, stronger responses on the next encounter. Weak stimuli, in contrast, tend to cause habituation; that is a weaker response on the next occasion [123]. During animal-assisted interventions, sensitization is a key factor to unsuccessful training and education, where animals are exposed to several stressors and cannot cope cognitively or emotionally [139], predisposing them to long-term behavioral issues and negative human-animal interactions [140].

### 7.7. Social Learning

Animals also have the capacity to learn by observing others; that is, with no need to live the experience, as occurs in asocial learning [141]. Social learning is more complex than the asocial form, but has been observed in insects [142], fish [143], birds [144], reptiles, amphibians [145], and mammals [146]. Monkeys, for example, attribute values to objects through observation, and then use those values to understand the decisions of other members of the group, specifically by observing feeding options. In this way, primates learn which foods are of higher value and more convenient without having to try them and fail [147,148]. In meerkats that live in open habitats, adults run to their hiding places and emit alarm calls in response to predators. The offspring react to this auditory cue and identify the threat by moving towards the closest adult. In his way, youngs learn the association between predators and certain alarm calls, to increase their rate of survival [149].

## 8. Motivation and Satiety

**Figure 6:**
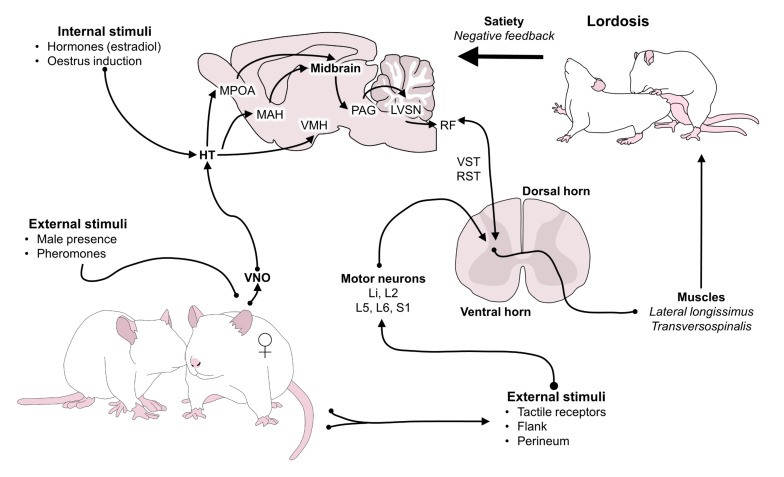
The mechanisms of motivation and satiety are complementary and mediate the appetitiveness or aversivity of stimuli. For example, a female rat may perceive a male as a positive stimulus. His odor triggers sexual motivation through the vomeronasal organ (VNO). The male produces positive feedback via tactile stimulation of the flanks and perineum. The valence of the odor and genital stimulation is processed in the medial amygdala, piriform cortex, and NAc, structures that project to the middle preoptic area (mPOA) and the anterior (MAH) and ventromedial hypothalamus (VMH), where appetitive sexual states are perceived. The information is projected to the midbrain (PAG), where the superior motor neurons convey it to the lumbosacral segments. The motor response involves centers of the lateral vestibular nucleus (LVSN) and reticular formation (RF). These axons reach the spinal cord through the lateral spinal vestibule (VET) and reticulospinal tract (RET) where, in conjunction with signals from tactile receptors, the contraction of the lateral longissimus and transverse spinal muscles are generated to produce lordosis [159]. HT: hypothalamus. After copulation, during satiety, the same olfactory and genital stimuli reduce their incentive value, and females will no longer experience reward. If they cannot get away, then copulation can evoke aversion.

Understanding what mechanisms energize animals to approach to, or withdrawal from certain stimuli is relevant to animal welfare. In general, the *seeking* system modulates states of motivation, desire, attention, and expectation. However, each of the seven basic neurobiological systems generate selectivity to respond to relevant stimuli. For instance, the *lust* system processes information to generate positive expectations of a socio-sexual encounter, which can be modulated by past experiences of sex. A virgin, sexually-naive rat may experience certain expectations through socialization and interaction with a mate that generated attraction due to its odor, but the virgin rat does not know the exact consequence of that interaction. By contrast, a sexually-experienced rat has the expectation of receiving a reward as a consequence of its own behavior. This is reflected in the speed and effort that mark the copulatory act. During the postcopulatory phase satiety mechanisms are triggered and the individual ceases to perceive those same stimuli as incentive. For example, after ejaculation, males show a reduced motivation by ceasing to court or pursue the female. First, when the rat detects a potential mate, its dopamine levels increase in structures like the NAc and continue to rise as proximity and sexual contact increase. However, at the moment of maximum sexual reward, dopamine in the NAc diminishes abruptly while other neuromodulators—opioids, serotonin, cannabinoids—increase to produce euphoria, satiety, and sedation, thus inhibiting sexual motivation [39]. In general, we can say that low-reward stimuli (like socializing with a receptive female) generate motivation to obtain more of the same thing, while high-reward stimuli (like sexual reward) tend to generate satiety and reduce motivation and desire. More exposure to an undesired stimulus may be experienced as aversive, which affects animal welfare (Figure 6). This mechanism occurs with every rewarding stimulus, like food, sex, socialization or a comfortable shelter. Eventually, all stimuli (despite they may be positive ones) may have the potential to turn into aversive ones if animals are not first allowed to built up motivation or desire to obtain such stimulus, namely, that animals must be exposed to “wanting” before they are exposed to “liking”.

Other examples of motivation include the flying behavior observed in some flocks of birds. Studies have demonstrated that this depends on endogenous opioids in the medial zone of the mPOA [148]. In fact, various behaviors derived from motivation, including feeding and grooming, respond to an increase of endogenous opioids and cannabinoids to regulate the internal concentrations of these substances [150]. In species like pigs, the behavior of wallowing in mud is a mechanism of thermoregulation that is reinforced because it allows the animal to lower its body temperature. In this case, motivation is closely linked to welfare, especially in animal production units. Referring to these systems, Elmore et al. [151] pointed to the relation between motivation and environmental enrichment in sows that showed a preference for an environment enriched with additional space for rooting. When animals are highly motivated but do not succeed in activating natural mechanisms of satiety they begin to manifest abnormal or stereotypical behaviors. These behaviors (although abnormal) trigger the release of opioids that reinforce their repetition. One example is the pacing observed in caged animals. A similar case comes from intensive water buffalo breeding systems. Like the offspring of all mammals, buffalo neonates are born with the motivation to suckle at their mothers’ udders [152], but separation from the dam prematurely interrupts this process and the young animals may develop abnormal behaviors like cross-suckling [153]. In reference to this, some authors mention that the development of stereotypical behaviors is linked to deficient feedback from a motivational state in which an animal fails to satisfy its needs completely and so remains in a state of high motivation or frustration. We can suggest that when mechanisms of satiety are not triggered then the *seeking* system continues its activity, generating frustration and abnormal expressions of “wanting”. For example, laying hens that are not provided with an adequate substrate for performing the behavior called dust-bathing—to eliminate ectoparasites and groom their feathers—may reflect this absence in reduced welfare that can potentially impact their productive parameters [154]. In commercial production systems of broilers and laying hens, this is a prevalent behavioral problem. To amend this, recent strategies towards animal welfare and environmental enrichment have been used peat or bales of lucerne hay to promote natural behaviors such as dust bathing, wing stretching, body shaking, and its usefulness in reducing lameness [155]. A similar result was observed by Baxter et al. [156], who found that approximately 22,000 broilers receiving oat hulls as dustbathing substrate showed better gait score and encouraged dust-bathing and foraging, but must be a part of a complementary enrichment program where other biological needs of the animals are covered. Furnished cages in 216 Hy-line brown layers have also shown that perches and dust-bath enrichment must consider social ranking since this can affect behavioral expression [157]. As Sandilands et al. [158] have shown, providing scratch mats to laying hens does not always have a beneficial effect on behavior or productive parameters, and the study regarding how to use the *seeking* system to provide welfare is still ongoing. One of the main reasons why this statement is true, is because when an animal is not provided with all the necessary elements to maintain a positive affective state, the presence or absence of behavioral systems responds to an emotional condition. If this is a negative one, it affects not only the zootechnical traits of the species but its mental and emotional state.

## 9. Brambell’s Five Freedoms and Neurobiology

Animal welfare has been traditionally ruled by the five freedoms of Brambell [160], which are meant to result in behaviors and physiological responses that indicate normality. Accordingly, well-being refers to freedom from: (1) hunger, thirst and malnutrition, (2) discomfort, (3) pain, (4) to express their natural behavioral repertoire, and (5) not exposed to fear and distress. The five freedoms of Brambell lay the foundations for animal welfare, but we must consider that well-being is a process, dependent of brain dynamics. Currently, animal welfare not only considers the five freedoms when applied to production, companion, wildlife, or laboratory species. In zoos, surveys have shown that zookeepers consider psychological factors to be more important than physical ones in zoo canids [161]. Recent studies have debated that it is not enough to ensure that animals do not suffer, they must experience positive emotional states to have good welfare [162]. The search for well-being through a vision that goes beyond solely avoiding negative states [163] promotes updating policies and innovation to provide adequate resources to animals to develop each emotional system [164].

## 10. Focusing on Emotional States

Generally speaking, the neurocircuits of this review are identified as either negative or positive, depending on the affective states they generate. Studies of the brain using electrical stimulation have demonstrated that the *fear*, *panic*, and *rage* systems generate negative emotions that feel bad as evidenced in animals that avoid self-stimulation of those regions [104,165]. In contrast, the *seeking*, *lust*, *care*, and *play* systems generate positive emotions because they support self-stimulation in animals [166]. The approach to, or avoidance of, electrical stimulation of those cerebral areas is interpreted as an objective measure of the degree to which those systems generate well-being or malaise [17]. For this reason, animal welfare strategies should be focused on fostering the activity of systems that produce positive affects and avoid (as possible) the activity of those systems that result in negative affects. The *seeking* system is stimulated when an animal explores, receives unexpected reinforcers, something sparks their curiosity and attention, or when animals are concentrated to obtain small reinforcers that keep them busy [2]. Likewise, both infants and adults should be offered opportunities to activate the *play* system, not only of the social type, but also motor play with novel objects. Social play is more likely to occur when two or more individuals are matched in size and age. It is important to understand that the *lust* and *care* systems are also generators of well-being, though it is obvious that in some enclosed conditions this cannot occur *ad libitum*. Finally, welfare programs should strive to diminish fear, rage, and panic. The latter may be the aspect that has received the least attention in zootechnical programs where the dams and offspring of various species are sold or separated for productive purposes. The *panic* system implies that the disruption of the social bond between individuals induces negative emotions such as sadness and depression, both in the dam and in the offspring [89]. Consequently, neuroendocrine signaling causes behavioral and physiological responses observed in animals. Because farm animals are social species, zootechnical programs with a welfare basis should prioritize social stability. Social support with conspecifics or family peers has been shown to improve their stress-coping ability, health, immune response and promote a positive psychological and physical state [165].

Since emotions can, indeed, be evaluated through behavior, it is essential to understand their varied expressions to intervene in benefit of individual animal welfare. For example, vocalizations emitted by species like dogs [167], cats [168], dairy cows [169], and piglets [170]. In this field, findings on facial expressions [171,172,173,174], and body postures are providing other important non-invasive tools that aid in evaluating animals’ emotions [175]. For instance, one study correlated the position of animals’ ears, neck, and tail in three distinct situations based on observations of differences in the position and movement of those body parts in response to different degrees of arousal and valence [176].

To date, evaluation of emotional states uses several behavioral items and elements such as cerebral lateralization that can reflect the activation of the right or left hemisphere and its association to the emotional processing of domestic and wildlife animals [177]. Moreover, the recognition that positive welfare includes more factors than just avoiding negative emotions has led to investigating the influence of different elements in the mental state of animals. Environmental enrichment, neuroendocrine response, and behavior can be used as a comprehensive indicator of positive welfare [178]. For example, in pigs subjected to play sessions with a toy, stability of peripheral levels of oxytocin, and tail movement behavior were associated with welfare [179]; or the activation of anticipatory behaviors in sheep subjected to positive stimuli (brushing) [180]. Therefore, the neuroscience of positive emotions in animals is a research field that needs to be addressed in-depth in future research.

## 11. Conclusions

Studying the neurobiology of behavior allows us to determine the whole range of stimuli that generate activity in neurobiological systems and their respective affective states and behaviors. This is of great importance for planning programs of welfare and environmental enrichment for animals in zoos and production units, as well as for pets. Analyzing animal behavior entails contemplating not only the observable motor responses that we as humans can perceive, but also understanding the biological mechanisms through which animals employ diverse neuronal connections, cerebral structures, and neuroendocrine systems, to perform specific behaviors and communicate.

## Data Availability

Not applicable.
